# Ketamine-induced prevention of SD-associated late infarct progression in experimental ischemia

**DOI:** 10.1038/s41598-024-59835-5

**Published:** 2024-05-03

**Authors:** A. Zdunczyk, L. Schumm, S. O. A. Helgers, M. Nieminen-Kelhä, X. Bai, S. Major, J. P. Dreier, N. Hecht, Johannes Woitzik

**Affiliations:** 1 Department of Neurosurgery, Charité-Universitätsmedizin Berlin, corporate member of Freie Universität Berlin, Humboldt-Universität zu Berlin, and Berlin Institute of Health, Berlin, Germany; 2 Center for Stroke Research Berlin, Charité-Universitätsmedizin Berlin, corporate member of Freie Universität Berlin, Humboldt-Universität zu Berlin, and Berlin Institute of Health, Berlin, Germany; 3 Department of Neurosurgery, Carl von Ossietzky University Oldenburg, Oldenburg, Germany; 4 Research Center Neurosensory Science, Carl von Ossietzky University Oldenburg, Oldenburg, Germany; 5 Department of Neurology, Charité-Universitätsmedizin Berlin, corporate member of Freie Universität Berlin, Humboldt-Universität zu Berlin, and Berlin Institute of Health, Berlin, Germany; 6 Department of Experimental Neurology, Charité-Universitätsmedizin Berlin, corporate member of Freie Universität Berlin, Humboldt-Universität zu Berlin, and Berlin Institute of Health, Berlin, Germany; 7 Bernstein Center for Computational Neuroscience Berlin, Berlin, Germany; 8 Einstein Center for Neurosciences Berlin, Berlin, Germany; 9 University Clinic for Neurosurgery, Marienstr. 11, 26121 Oldenburg, Germany

**Keywords:** Spreading depolarization, Ketamine, Stroke progression, Experimental ischemia, Neuroscience, Preclinical research, Stroke

## Abstract

Spreading depolarizations (SDs) occur frequently in patients with malignant hemispheric stroke. In animal-based experiments, SDs have been shown to cause secondary neuronal damage and infarct expansion during the initial period of infarct progression. In contrast, the influence of SDs during the delayed period is not well characterized yet. Here, we analyzed the impact of SDs in the delayed phase after cerebral ischemia and the potential protective effect of ketamine. Focal ischemia was induced by distal occlusion of the left middle cerebral artery in C57BL6/J mice. 24 h after occlusion, SDs were measured using electrocorticography and laser-speckle imaging in three different study groups: control group without SD induction, SD induction with potassium chloride, and SD induction with potassium chloride and ketamine administration. Infarct progression was evaluated by sequential MRI scans. 24 h after occlusion, we observed spontaneous SDs with a rate of 0.33 SDs/hour which increased during potassium chloride application (3.37 SDs/hour). The analysis of the neurovascular coupling revealed prolonged hypoemic and hyperemic responses in this group. Stroke volume increased even 24 h after stroke onset in the SD-group. Ketamine treatment caused a lesser pronounced hypoemic response and prevented infarct growth in the delayed phase after experimental ischemia. Induction of SDs with potassium chloride was significantly associated with stroke progression even 24 h after stroke onset. Therefore, SD might be a significant contributor to delayed stroke progression. Ketamine might be a possible drug to prevent SD-induced delayed stroke progression.

## Introduction

Ischemic stroke, driven by an increasing incidence due to the aging population, stands as one of the leading causes for disability and mortality worldwide^1^. Despite the development of elaborated neuroprotective strategies aimed at mitigating the risk for secondary stroke progression, lesion progression remains the primary complication in these patients^2^.

In this context, spreading depolarizations (SDs) have gained an increase in attention as the neuronal and astroglial process that precedes neuronal death, both in the core of ischemia^3–7^ and the ischemic penumbra^8,9^. Spreading depolarizations are characterized by a wave of near-complete breakdown of the transmembrane ion gradients and cytotoxic edema as well as sustained neuronal and astroglial mass depolarization traveling across the cortex and other gray matter structures^10^. Importantly, even in the ischemic core, SD is always an initially reversible process. This means that neurons can survive SD even in the ischemic core if the tissue is reperfused and repolarizes before the so called commitment point^3,7,11,12^.

Sustained mitochondrial depolarization, glutamate release, and intracellular increases in neuronal sodium and calcium levels are discussed as factors responsible for the transition from the initially reversible SD component to the negative ultraslow potential (NUP), indicating the development of cell death in the ischemic core^3,13,14^. The same factors are also discussed for SD-induced damage progression in the ischemic penumbra, in which the transition from SD to NUP may also occur, albeit after a longer latency period and after shorter SDs have already occurred^15–17^. Whether shorter duration SDs in the penumbra that do not show transition to the NUP also contribute to damage progression is more controversial^18^. SDs originating from the ischemic zone and invading the surrounding well-perfused tissue might even have beneficial effects on the surrounding tissue^5,19^. In all this, it is important to remember that the same wave can behave biologically differently at different locations through which it passes, i.e., the same SD wave can cause harm at one location but not at another^4^. Given the Janus-faced nature of SD as a mechanism of harm when it is locally long-lived, and as a harmless or even potentially beneficial factor when it is locally short-lived, the different hemodynamic responses to SDs are of particular interest from a mechanistic perspective because they strongly influence the local duration of SD.

In phylogenetically more evolved mammals such as rat, swine and human, the hemodynamic response to SD is characterized by a transient hyperperfusion followed by oligemia in healthy tissue^20–22^. In contrast, in tissues with impaired neurovascular machinery, SD shows the inverse hemodynamic response, characterized by severe hypoperfusion (spreading ischemia) during the neuronal depolarization phase, which is often followed by hyperemia, depending on the exact conditions^5,23–25^. Importantly, spreading ischemia impedes the local recovery from SD. Therefore, the SD and hence the cytotoxic edema become prolonged and the likelihood of local tissue injury increases^5,26^. In the ischemic core and penumbra, SDs typically induce inverse hemodynamic responses exacerbating preexisting ischemia^3,4,17,25,27^. As in other species, preexisting ischemia further shifts the hemodynamic response toward predominant vasoconstriction (inversion) in mice^4,28^.

Following this evidence, in experimental stroke models, the quantity and duration of SDs proved to be correlated with an enlarged infarct size in the early phase (< 24 h) following stroke induction^3,8,9,28–31^. The association of delayed SDs with lesion progression was also found in a recent study in patients with malignant hemispheric stroke (Kowoll et al., unpublished data).

To counteract the occurrence of SDs and to potentially prevent lesion progression, various pharmacological targets have been discussed. In this context, the non-competitive antagonist of the ionotropic N-methyl-D-aspartate receptor (NMDAR) ketamine has been studied. High-dose ketamine improved neurological outcome following experimental cerebral ischemia and reduced neuronal cell death after global forebrain ischemia in rats^30,32,33^. In this context, it is interesting that ketamine has been found to inhibit SDs experimentally and clinically^34–40^. Even though the exact mechanism that leads to inhibition of SDs is not known yet, it is hypothesized that ketamine protects the cell membrane from mass depolarization by stabilizing the glutamate binding side^40^.

Most experimental studies investigated the acute phase of cerebral ischemia. Since the risk of infarct progression can persist for several days, the later phase of cerebral ischemia (> 24 h) also holds clinical significance. During this period, patients are treated in the hospital and secondary lesion progression is thus potentially modifiable. A first report by Schumm et al. 2021 showed that SDs, induced by KCl in the delayed phase after experimental ischemia, promote infarct progression. In order to investigate the effect of ketamine on the role of SDs in lesion progression during the later phase of experimental cerebral ischemia, in the current study we analyzed SD occurrence and neurovascular response to SDs after distal middle cerebral artery occlusion (dMCAO) in presence and absence of low-dose ketamine treatment.

## Material and methods

### Experimental subjects and study design

Male C57BL6/J mice (n = 30, Charles River Laboratories) with an age of 12–15 weeks and a weight of 28–33 g were used. All animals were kept in an enriched environment with unlimited access to food and water. During the entire experimental phase, animals were continuously monitored for their well-being. All experimental procedures were conducted in the same order and manner across all experimental groups to avoid confounders and achieve high level standardization.

**Figure 1:**
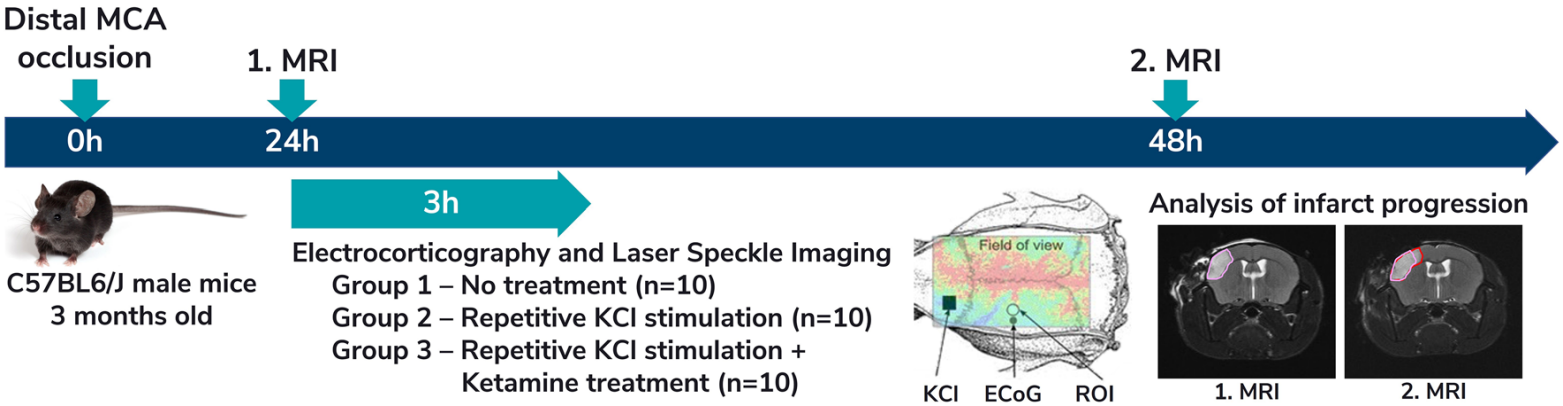
Schematic representation of the study design including the temporal course of the experiment and the different experimental groups. KCl—potassium chloride application location; ECoG—electrophysiological recording location in the penumbra region; ROI—region of interest used for laser speckle imaging analysis.

At the beginning of the experimental procedure (Fig. 1), ischemic stroke was induced in all animals by occlusion of the dMCAO as described in Schumm et al. 2021. In brief, animals were anaesthetized by intraperitoneal injection of ketamine/xylazine (80 mg/kg; 16 mg/kg), a left sided cranial window was prepared, and the left distal MCA was completely and permanently electrocoagulated. Paracetamol enriched drinking water (300 mg/kg) and local Xylocain gel was used as analgesia.

24 h after stroke induction all animals were subjected to magnetic resonance imaging (MRI; 7 T rodent scanner (Pharmascan70/16US, Bruker Biospin, USA) T2-weighted 2D turbo spin-echo sequence) as described before^41,42^.

The MRI was followed by a three-hour electrocorticographic (ECoG) recording of SDs. Simultaneously, regional CBF was measured by laser speckle imaging (LSI). Animals were randomly assigned into three different experimental groups: In group 1 (n = 10; control group) the spontaneous occurrence of SDs was measured without further interventions. In group 2 (n = 10; KCl group) SDs were repetitively induced every 15–20 min through KCl application. In group 3 (n = 10; ketamine group) SDs were repetitively induced every 15–20 min by KCl while the animals received ketamine.

A second MRI was performed in all animals 48 h after stroke induction to evaluate lesion progression. After the second MRI, all animals were killed by decapitation under deep anaesthesia with intraperitoneal injection of ketamine/xylazine (80 mg/kg; 16 mg/kg).

### Electrocorticography and laser speckle imaging

After the first MRI measurement (24 h after stroke induction) all animals were subjected to a three-hour ECoG recording. The anaesthesia protocol consisted of a low-dose inhalative isolflurane (2–3% induction dose, < 1% maintenance dose) with maintained spontaneous breathing and continuous body core temperature (rectal probe) with a feedback-controlled heating mat. Depth of anaesthesia was monitored through respiratory rate measured by a pressure sensor (ADinstruments, New Zealand) on the animal’s chest. In the ketamine group, ketamine (25 mg/kg) was administered intraperitoneally every 45 min throughout the recording period.

The skull was exposed, and two left hemispheric burr holes with a diameter of 2 mm were drilled. The first burr hole was located 2 mm lateral and ventral to Bregma. It was used for the repetitive SD induction by application of a potassium chloride (KCl, 1 M) soaked cotton ball (1 mm) for maximally 5 min. The second burr hole was located over the left parietal cortex above the penumbra region determined by LSI for insertion of the ECoG-electrode. Both burr holes were drilled under continuous saline cooling and preservation of the dura.

SD associated potential changes (DC/AC-ECoG) on the cortical surface were recorded by an Ag/AgCl glass microelectrode with a tip diameter of 3 µm, inserted through the burr hole above the penumbra into the parietal cortex at a depth of 200 µm. The Ag/AgCl reference electrode was placed under the nuchal skin. Data was sampled continuously at 5 Hz (LabChart Version 8; all ADInstruments, New Zealand) and amplified (FE231 Bridge Amplifier) and digitized at 100 Hz (16/35 PowerLab).

Simultaneously to the ECoG recordings, hemodynamic changes were measured by LSI (moFLPI-1, Moor Instruments Ltd., Axminster, UK;^43,44^). In order to achieve a high temporal resolution, the image acquisition frequency was set to 5 Hz. The spatial resolution was 152 × 113 pixels. Cortical perfusion was calculated in the arbitrary perfusion unit CBF-Flux. For analysis, a region of interest with a diameter of 0.36 mm was placed in the penumbra zone adjacent to the tip of the microelectrode.

### Data analysis and statistics

ECoG data was analyzed with LabChart (v8, ADInstruments, New Zealand) in accordance to the COSBID guidelines^10^. SD-duration (in minutes) and -amplitude (in mV) were calculated from the DC-ECoG signal (0.01–45 Hz). Depression time was assessed using the AC-ECoG (0.5–45 Hz).

Hemodynamic responses were analyzed in the penumbra region for each SD. The amplitude (lowest and highest level of hypo- and hyperperfusion compared to baseline in %) and duration (time until the rCBF reached baseline after a hypo- or hyperperfusion in minutes) of the hemodynamic response was measured.

Lesion progression was determined based on MRI comparing T2-weighted and edema corrected lesion volumes 24 h and 48 h after dMCAo (see^45^ and^41^ for detailed technical description).

Data was analyzed in a blinded fashion. Outlier detection was performed on all data and excluded from further analysis according to Grubb’s test method. Experimental groups were compared using a one-way ANOVA (Tukey’s multiple comparisons post-hoc test). The level of significance was set to p < 0.05 (two-sided). Data are shown as mean ± standard deviation including original data points. Statistical analysis and plotting were performed with Prism (v9, GraphPad software, San Diego, CA, USA).

### Ethical approval

All animal experiments were approved by the local committee of health (Landesamt für Gesundheit und Soziales (LAGeSo), Berlin, G0156/15) and performed according to the National Animal Welfare Act and the Charité Animal Welfare Guidelines. The animals were kept and treated according to the Guide for the Care and Use of Animals (National Research Council) and the EU Directive 2010/63/EU. Reporting was accomplished according to the Animal Research: Reporting of In Vivo Experiments (ARRIVE) guidelines.

## Results

### Occurrence of spreading depolarizations

**Figure 2:**
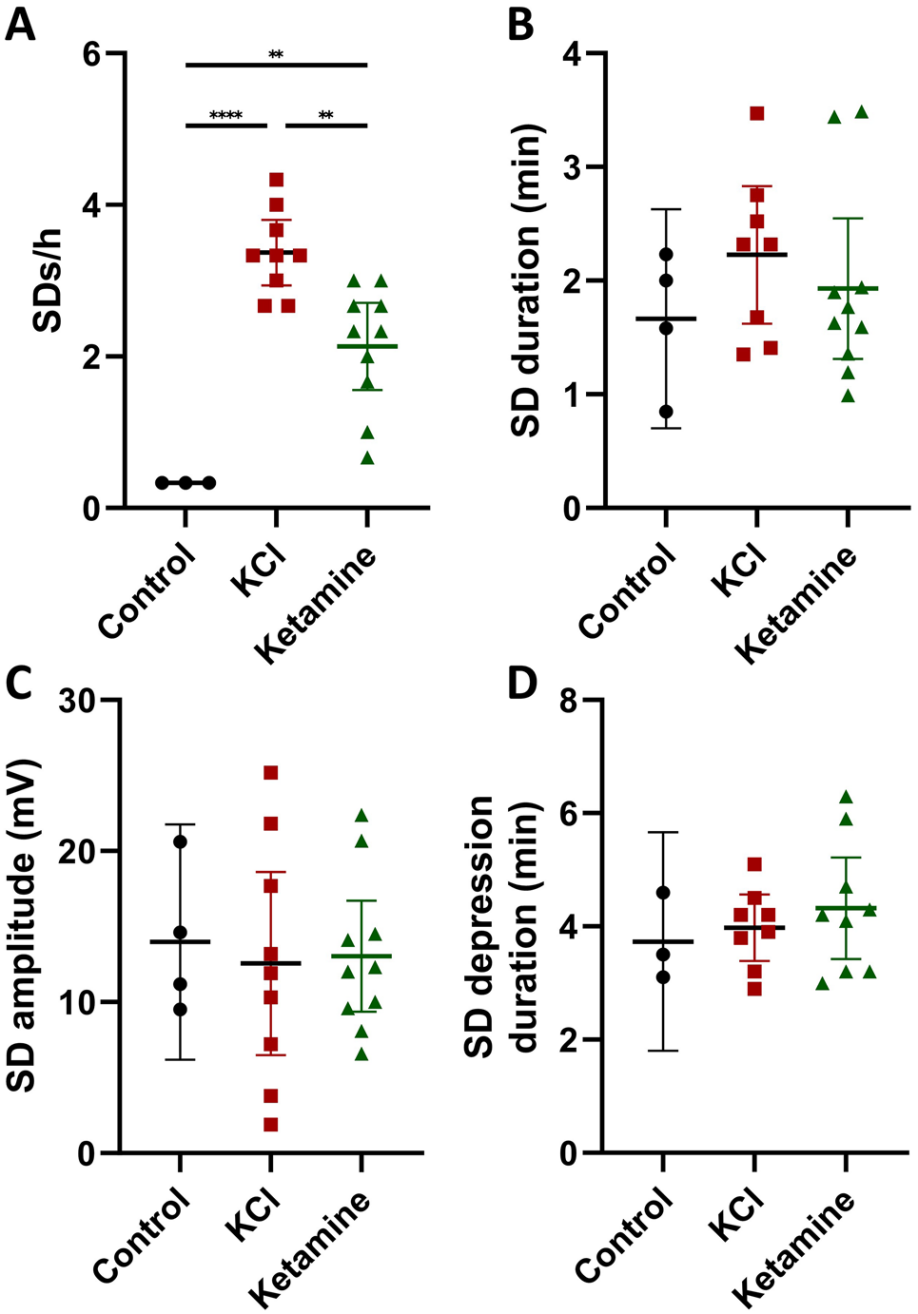
Occurrence (SDs per hour; **A**), duration (minutes; **B**), amplitude (mV; **C**) and depression duration (minutes; **D**) of the observed SDs during the electrocorticographic recordings. Data is shown as individual symbols and mean ± standard deviation (** p ≤ 0.01; *** p ≤ 0.001).

The occurrence of SDs in the penumbra 24 h hours after dMCAO varied substantially across experimental groups during the three-hour recording phase. In the control group (n = 10), in which the spontaneous occurrence of SDs was monitored, SDs were detected in only 3 animals with a frequency of 0.33 SDs/hour (Fig. 2A). The repetitive application of a KCl soaked cotton ball on the cranial burr hole (n = 9, one animal was excluded from analysis because of the occurrence of a second infarct independent from the experimental procedures) significantly increased the SD occurrence compared to the control group to 3.37 ± 0.56 SDs/hour (n = 9; F_(2,19)_ = 24.98, p < 0.0001; post-hoc p < 0.0001; Fig. 2A). In the animals that received ketamine before and during the KCl stimulation period, a rate of 2.13 ± 0.80 SDs/hour was observed (Fig. 2A). As for the KCl group, statistical analysis revealed a significant increase in SDs/hour compared to the control group (n = 10; F_(2,19)_ = 24.98, p < 0.0001; post-hoc p = 0.0016), however additionally a significant decrease compared to the KCl group (n = 10; F_(2,19)_ = 24.98, p < 0.0001; post-hoc p = 0.0019).

In contrast to the SD frequency, the mean duration was not significantly different between the groups. The mean SD duration in the control group was 1.67 ± 0.61 min (n = 4), in the KCl group 2.23 ± 0.72 min (n = 8) and in the ketamine group 1.93 ± 0.86 min (n = 10, Fig. 2B). The cumulative SD duration in the KCL group was, due to the significantly higher number of SDs in this group, significantly increased compared to the control group (n = 9; F_(2,18)_ = 17.00, p < 0.0001; post-hoc p = 0.0002) as well as compared to the ketamine group (n = 9; F_(2,18)_ = 17.00, p < 0.0001; post-hoc p = 0.0009).

In addition, the amplitude of the DC-shift (Fig. 2C) and the depression duration in the AC-ECoG (Fig. 2D) was analyzed. No differences were found for either of the parameters between the experimental groups.

### Neurovascular response to SD

**Figure 3:**
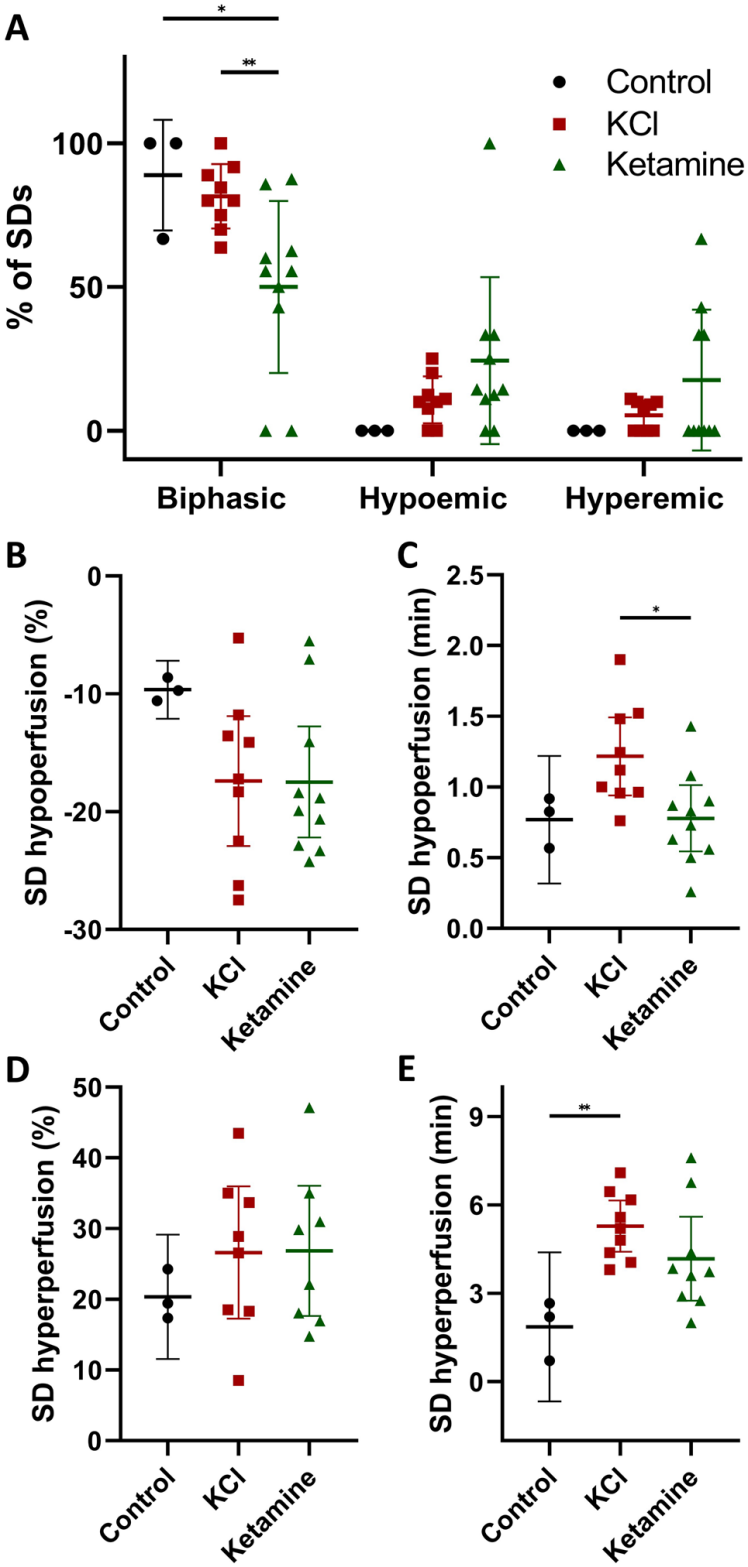
Neurovascular coupling of the SDs measured by Laser Speckle Imaging during the 3-h recording phase. Categorization of the recorded SDs depending on their neurovascular response (biphasic, hypoemic, hyperemic) in % of the total number of SDs in the respective study group (**A**). Amplitude (% compared to baseline) and duration (minutes) of the hypoemic (**B**, **C**) and the hyperemic (**D**, **E**) neurovascular response after SD inductions. Data is shown as individual symbols and mean ± standard deviation (* p ≤ 0.05; ** p ≤ 0.01).

The analysis of the neurovascular response to SD revealed solely biphasic CBF responses in the control group, whereas in the other experimental groups also pure hypoemic and hyperemic responses were observed (Fig. 3A). In the KCl group, the proportion of non-biphasic responses (hypoemic: 10.64% and hyperemic: 5.32%) was smaller compared to the biphasic CBF responses (84.04%). In the ketamine group, the number of biphasic responses (62.72%) was significantly decreased compared to the other experimental groups (n = 10; F(2,57) = 54.48, p < 0.0001; post-hoc p = 0.0140). The non-biphasic responses in the ketamine group were divided into 20.34% solely hypoemic and 16.94% hyperemic reactions.

The amplitude of the hypoemic response was − 9.64 ± 0.99% ΔrCBF (n = 3) in the control group, − 17.39 ± 7.17 %ΔrCBF (n = 9) in the KCl group and − 17.48 ± 6.59 %ΔrCBF (n = 10) in the ketamine group (Fig. 3B). Even though there was a stronger hypoemic response in the KCl and the ketamine group, statistical analysis showed no significant difference between the groups. In contrast, the duration of the hypoemic response to SD was significantly shorter in the ketamine group (0.78 ± 0.33 min; n = 10) compared to the KCl group (1.22 ± 0.36 min; n = 9; F(2,19) = 4.767, p = 0.0210, post-hoc p = 0.0238; Fig. 3C). Neither the ketamine nor the KCl group showed a significant difference compared to the control group (0.77 ± 0.18 min, n = 3; Fig. 3C).

A similar pattern as for the hypoemic response was observed for the amplitude and duration of the hyperemic response. Regarding the amplitude, there was a non-significant difference between the control group (20.36 ± 3.54 %ΔrCBF; n = 3) and the two other experimental groups (KCl-group: 26.62 ± 11.17 %ΔrCBF (n = 8); Ketamine group: 26.86 ± 11.00 %ΔrCBF (n = 8); Fig. 3D). The duration of the hyperemic response was significantly increased in the KCl group (5.28 ± 1.14 min, n = 9) compared to the control group (1.87 ± 1.02 min; n = 3; F(2,18) = 6.01, p = 0.01; post-hoc p = 0.0077; Fig. 3E). Interestingly, ketamine treatment slightly reduced the duration of the hyperemic response (4.18 ± 1.86 min; n = 9) compared to the KCl group, but showed no significant difference to neither of the other experimental groups (Fig. 3E).

### Infarct progression

**Figure 4:**
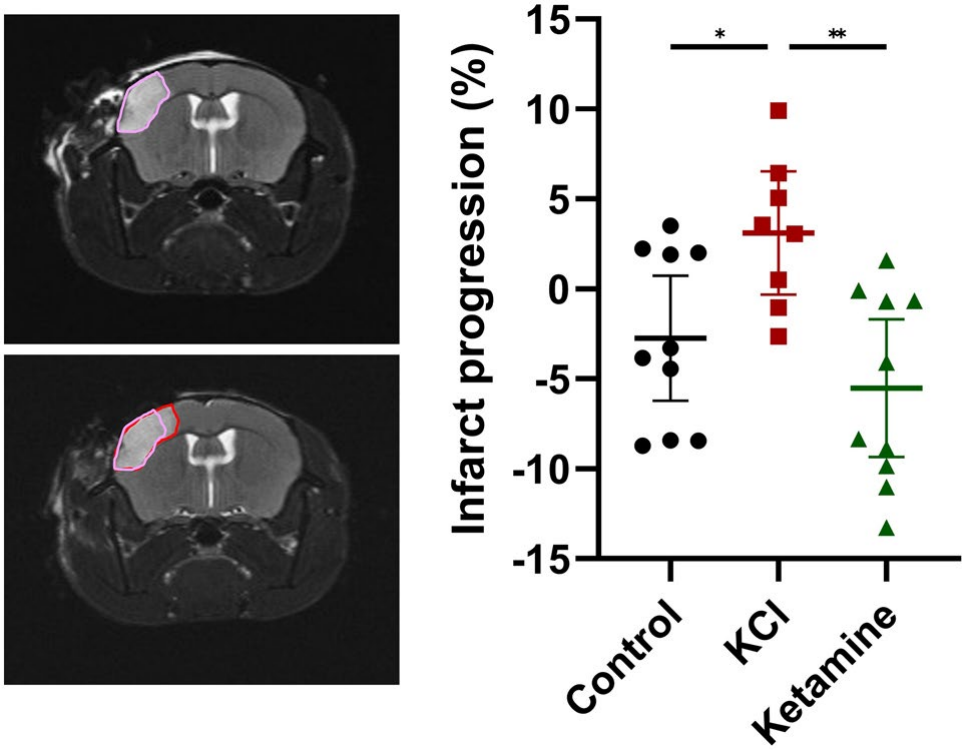
Representative example of MRI measurements 24 and 48 h after stroke induction. Infarct size and growth is depicted by the colored areas. Analysis of the infract progression between the first and second measurement in %. Data is shown as individual symbols and mean ± standard deviation (* p ≤ 0.05; ** p ≤ 0.01).

Infarct progression was measured by comparing the lesion volumes 24 h and 48 h after dMCAO. In the control group, no lesion progression, but rather a slight decrease in lesion size of − 2.74 ± 4.86% (n = 10) was observed. In contrast, there was a 3.11 ± 4.11% increase in lesion size on the second MRI in the KCl group (Fig. 4). One-way ANOVA revealed a significant increase in lesion progression compared to the control group (n = 10; F(2,25) = 7.196, p = 0.0034; post-hoc p = 0.0445). Interestingly, in the group which was treated with ketamine in addition to the KCl-stimulation, the infarct size decreased between the two MRI measurements by − 5.53% (n = 10; Fig. 4). One-way ANOVA revealed a significant decrease in lesion progression compared to the KCl group (n = 10; F(2,25) = 7.196, p = 0.0034; post-hoc p = 0.0026) but no difference to the control group.

## Discussion

This study aimed to explore the effect of low-dose ketamine treatment on the occurrence of SDs and neurovascular responses to SDs, and their impact on the infarct evolution during the delayed phase of experimental cerebral ischemia. In the control group, sporadic spontaneous SDs were observe even 24 h hours after dMCAo without further manipulations. This finding aligns with clinical and experimental studies, indicating that SDs occur not only in the acute phase but, with a decreased incidence, also in the delayed phase after ischemia^29,41,46^. These delayed SDs were associated with solely biphasic blood flow responses. On the follow up MRI 48 h after stroke onset, no lesion progression was detected. Following the hypothesis that the occurrence, and especially the duration, of SDs may cause stroke progression, the very low number of spontaneous SDs and their comparably short duration in the ECoG and the LSI might have had a beneficial influence.

As expected, the number of SDs increased in the KCl group. Additionally, the vascular response showed a tendency towards stronger hyper- and hypoemic CBF responses following SDs. The enhancement of hypoemic responses to SD by an increase in extracellular potassium concentration is well compatible with experiments in the rat in which artificial cerebrospinal fluid with increased potassium concentration was applied locally to the cortex^47^. Furthermore, the duration of the SD-associated CBF response was prolonged in the KCl group compared to the control group with spontaneous SDs. This also agrees with earlier studies and is probably explained by the fact that potassium prolongs the depolarization phase of the SDs, which has already been demonstrated in rat brain slices^48^.

The follow-up MRI after 48 h revealed a significant increase in infarct size in the KCl group compared to the control group. Even though the increase in infarct size is relatively small (3.11 ± 4.11%), it is still noteworthy as the main infarct maturation occurs within the initial 24 h after vessel occlusion^41,49–51^. During the subsequent period, the estimated infarct volume based on MRI scans without interventions tended to decrease in size, as seen in the control group^52,53^. Therefore, as shown by Schumm et al.^41^, the repetitive induction of SDs seems to prolong the period of stroke induced permanent damage. These findings support the hypothesis of a pathophysiological role of SDs in delayed lesion progression after ischemic stroke. In particular, the pronounced and prolonged hypoemic response might have decreased the energy supply in the metabolically compromised tissue of the penumbra^6,10^.

Even though the association of SDs and delayed infarct progression has been shown multiple times, this causative role has been questioned by optogenetic studies in which repetitive SD induction did not worsen acute ischemic stroke outcome^18,54^. It has been proposed that SDs rather serve as a biomarker than as the cause of the infarct progression. This is in line with clinical studies, in which SD, and especially the cumulative SD-duration, is identified as an independent biomarker of progressive brain injury in subarachnoid hemorrhage patients^55^. In the current study, the cumulative SD duration was significantly increased in the KCl group. This result is in line with the clinical observation, as infarct progression is closely related to neurological outcome. However, as the mean SD duration was the same across all experimental groups, this increase can be explained by the significantly larger total number of SDs during the measuring period and thereby does not reflect a change in the electrophysiological characteristics of the single SDs.

To assess the role of NMDA antagonists as a possible therapeutic treatment option, animals received low dose ketamine (25 mg/kg BW i.p.) during the three-hour recording period. The treatment led to a significantly lower number of SDs compared to the KCl group and a significantly higher number compared to the control group. Interestingly, the amplitude of the hemodynamic response to the SDs was similar to the KCl group, whereas especially the duration of the hypoperfusion significantly decreased. The second MRI after 48 h indicated a significant decrease in lesion size compared to the KCl group.

Ketamine has been discussed before as a possible therapeutic agent in the acute phase of ischemic stroke and has recently been shown to be safe, despite possible side effects like increased intracranial pressure^56–58^. Furthermore, ketamine's ability to reduce or block SD occurrence has been demonstrated, along with its perfusion-promoting effects^36,40^. Even though the blockade of SDs has been shown to be most effective for SDs originating in metabolically intact tissue, the effect might be of relevance for the periinfarct area, as these SDs might invade this area and cause spreading ischemia promoting infarct progression^36^.

Experimentally, ketamine has been shown to mitigate the harmful consequences in peri-infarct tissue during the acute phase^34,35^. Consistent with these results, our study indicates that even the administration of low-doses of ketamine in the delayed phase after experimental ischemia is able to antagonize lesion progression by preventing SD-associated secondary lesion progression.

Based on the current data, a direct causality between the occurrence of SDs and delayed infarct progression cannot be deducted. The induction of SDs using KCl might lead to direct tissue injuries and therefore confound the results of the enlarged infarct volume^13,18,59,60^. However, in the ketamine group, no infarct progression was observed, justas in the control group which received no KCl. Furthermore, as the KCl induction was performed distant to the infarct, direct damage due to KCl itself would have been expected at the induction side, which was, at least in the MRI after 48 h, not observed in the current study.

An additional potentially influencing factor on neuroprotection, is the used isoflurane anesthesia which has been shown to be able to model tissue oxygenation and thereby reduce SD-associated hypoxia^61^. However, as the anesthetic protocol was the same in all groups, the influence should be equally across the experimental groups.

Due to the short observational period in this study, the results do not fully encompass the entire process of lesion progression after experimental ischemia. For example, perfusion-dependent impairment in cerebral autoregulation was not assessed in the current experiments^62^. Additionally, SD recording was only possible in the three-hour recording phase. Since the number of SDs appears to influence infarct progression, the actual number of spontaneously occurring SDs could explain the relatively large variation in infarct progression across different study groups. Besides the infarct size, inclusion of neurological outcome parameters would enhance the translational relevance, especially for the possible beneficial effects of ketamine.

Our results highlight the occurrence and influence of SDs during a subacute stage of experimental stroke. By increasing the number of SDs, recovery of tissue at risk seems to be limited, which results in enlarged infarct sizes even 48 h after stroke onset. Administration of low-dose ketamine prevented this stroke progression by reducing the SD numbers and shifting neurovascular coupling towards shorter hypoemic responses. Therefore, ketamine might be a possible agent counteracting the potential damaging effect of SDs in the delayed phase after stroke induction.

## Data Availability

Data is available upon reasonable request from the corresponding author.
